# Obesity in Tanzanian Youth (15–35 Years): From Nutrition Transition to Policy Action—A Scoping Review

**DOI:** 10.3390/nu18010061

**Published:** 2025-12-24

**Authors:** Angeliki Sofroniou, Sara Basilico, Maria Vittoria Conti, Haikael David Martin, Hellas Cena

**Affiliations:** 1 Department of Food and Drug, University of Parma, 43124 Parma, Italy; angeliki.sofroniou@unipr.it; 2 Laboratory of Dietetics and Clinical Nutrition, Department of Public Health, Experimental and Forensic Medicine, University of Pavia, 27100 Pavia, Italy; mariavittoria.conti@unipv.it (M.V.C.); hellas.cena@unipv.it (H.C.); 3 School of Life Sciences and Bioengineering, Nelson Mandela African Institution of Science and Technology, Arusha, Tanzania; haikael.martin@nm-aist.ac.tz; 4 Clinical Nutrition Unit, Istituti Clinici Scientifici Maugeri Istituto di Ricovero e Cura a Carattere Scientifico, 27100 Pavia, Italy; 5 Italian Institute for Planetary Health, 00168 Rome, Italy

**Keywords:** Tanzania, low-middle income countries, obesity, nutrition transition, food policies, youth

## Abstract

Background: Tanzania is undergoing a rapid nutrition and epidemiological transition that has shifted dietary patterns and lifestyles toward more Westernised models, contributing to an increase in diet-related non-communicable diseases (NCDs), including obesity. Youth aged 15–35 years are particularly vulnerable to these shifts. Objectives: The objective of this scoping review was to map the available evidence on youth obesity in Tanzania, focusing on (1) data gaps in epidemiological reporting; (2) the ongoing nutrition transition; and (3) existing food system and health-related policies targeting youth. Methods: A targeted search was conducted in PubMed, Scopus, and the grey literature. The PCC (Population/Concept/Context) framework guided the study selection, focusing on youth and general young adults aged 15–35 years in Tanzania. Eligible studies published between 2000 and June 2025 were included. Results: The search yielded 247 peer-reviewed articles, of which 35 met the inclusion criteria. The findings reveal substantial gaps in epidemiological reporting, particularly limited regional data and inconsistent age disaggregation, which often obscures youth-specific patterns. Evidence on nutrition and lifestyle transitions is limited and fragmented, while available policies addressing obesity and related risk factors are broad in scope and rarely tailored to the youth population. Conclusions: This review demonstrates that evidence on obesity among Tanzanian youth is scarce, unevenly reported, and insufficiently specific to this age group. Clear gaps exist in epidemiological surveillance, research on nutrition transition, and youth-focused policy design. Strengthening age-specific monitoring systems, generating context-specific evidence, and developing targeted, measurable, and actionable strategies for youth could enhance Tanzania’s efforts to curb the rising burden of obesity and related NCDs.

## 1. Introduction

Tanzania, like many East African countries, faces a triple burden of malnutrition, encompassing undernutrition (e.g., stunting and underweight), micronutrient deficiencies, and overnutrition (e.g., overweight and obesity) [1]. These conditions collectively contribute to the growing burden of diet-related non-communicable diseases (NCDs) [2,3]. Across East Africa, NCDs have risen sharply, with a reported 67% increase over the past 30 years, and the prevalence of obesity is increasing at an even faster rate than the associated disease burden [4]. While undernutrition has historically been the predominant concern in sub-Saharan Africa, overweight and obesity have emerged more recently as significant and rapidly growing public health challenges.

In parallel, a nutrition transition is being observed where traditional diets are being exchanged for high-energy foods with low nutritional profile, resulting in inadequate dietary intakes [5]. Nutrition transition coincides with economic changes, and it is suggested to also result from an epidemiological transition [6,7]. Low-income countries experiencing economic development start overcoming communicable diseases, one of the immediate causes of malnutrition, with the provision of clean water, medicines, and better public health services. With economic growth, governments can provide better food security and food access. At the same time, people also move to urban areas for employment and start having more disposable income. In Tanzania, there has been a significant growth of the middle class [8], characterised by a nutrition transition to a Western-pattern diet and sedentary behaviour. These changes lead to an increase in NCDs, including obesity, diabetes, and cardiovascular disease [9].

Although some of the underlying causes of malnutrition are being addressed to bridge the gap between its basic and immediate causes, the latter persist but are reversed; communicable diseases are substituted by NCDs and undernutrition is by overnutrition [10]. Common modifiable risk factors that underlie major NCDs and allow for windows of opportunity for intervention include tobacco, alcohol abuse, raised blood pressure, blood sugar and cholesterol, unhealthy diets, insufficient physical activity, and overweight/obesity, with the latter being the most prevalent modifiable risk factors globally. Hence, the underlying causes of malnutrition should be addressed by first strengthening fundamental factors such as economic and political structures, resource growth and control, and education and training of human capital. At the heart of this transformation are young generations, who drive innovation and progress and represent the future of their nations. In this context, we adopted the African Youth Charter’s definition of youth (15–35 years) for Sub-Saharan Africa [11], as this age group undergoes a critical transition marked by increasing autonomy, economic independence, and exposure to socioeconomic risk factors for obesity. Poor dietary habits, including skipping meals, eating out, and social influences on food choices, further contribute to the rising burden of overweight and obesity in this population [12]. Strengthening nutrition and education among young people is essential, not only for improving their health but also for breaking the intergenerational cycle of malnutrition and fostering healthier future generations.

### 1.1. Scoping Review Research Question

Obesity among youth aged 15–35 years is an increasingly pressing public health issue in the United Republic of Tanzania. Given the fragmented and heterogeneous nature of the available evidence, spanning epidemiological data, nutrition transition dynamics, and policy responses, a scoping review was considered the most appropriate methodological approach. This method allows for a comprehensive mapping of existing studies, the identification of knowledge gaps, and an integrated understanding of how these interconnected domains shape the emerging obesity burden among Tanzanian youth.

To provide this broad and structured overview, the review addressed the following specific questions:

1. What evidence exists on the prevalence and determinants of obesity among youth in Tanzania, and what are the key data gaps?
2. What evidence describes the ongoing nutrition transition, including reported dietary and lifestyle changes among youth?
3. What national or local policies address food systems, physical activity promotion, and public health education targeting youth obesity in Tanzania?

### 1.2. Scoping Review Objectives

Following these research questions, the main objective of this review was to map and synthesise the available evidence on obesity among youth aged 15–35 years in Tanzania. More specifically, the review aims to describe the existing evidence, and the remaining gaps, on the prevalence and determinants of youth obesity; to explore the literature on the ongoing nutrition and lifestyle transition contributing to rising obesity rates in this age group; and to identify national and local policies related to food systems, physical activity promotion, and public health education targeting youth.

Building on these findings, the review also provides evidence-informed policy recommendations derived from the Tanzanian context and from comparable settings.

## 2. Materials and Methods

This scoping review was conducted using the Preferred Reporting Items for Systematic Reviews and Meta-Analyses (PRISMA) for scoping review [13], and framework by Joanna Briggs Institute (JBI) [14]. This review was not registered in the Prospective Register of Systematic Reviews (PROSPERO) since it is a scoping review and not a systematic review. Before developing this scoping review, a literature search was conducted on PubMed to identify any previously published exploratory reviews on obesity, nutritional transition, and policies in Tanzania among the youth (15–35-year-olds). No previously published studies on this topic were identified.

### 2.1. Search Strategy

PubMed and Scopus were searched to retrieve articles published in the last 25 years (2000–2025). Accordingly, only studies meeting the eligibility criteria discussed below were included in the review. The initial search was conducted on 26 June 2025 and an updated search on 3 July 2025. The following search terms and keywords were used as follows: “obesity”, “overweight”, “prevalence”, “Tanzania”, “nutrition transition”, “dietary consumption”, “policy”, “food policy”, “physical activity”, and “education campaigns” and their relevant components and/or synonyms. All the above searches were complemented with searches of the grey literature using official government websites such as the United States Agency for International Development (USAID), The United Republic of Tanzania Ministry of Health, or websites of intergovernmental organisations including Food and Agriculture Organization (FAO) and World Health Organization (WHO).

An elimination process was conducted (based on title, abstract, key terms, and full articles in ascending order) by two independent researchers. The results were compared and consolidated through consensus between the two researchers in July 2025. A third in-dependent researcher further reviewed the final list of included articles. Any disagreement on the final papers to be included was discussed until all reviewers reached an agreement.

### 2.2. Eligibility Criteria

#### 2.2.1. Inclusion Criteria

Since this is a scoping review, we used the PCC (Population/Concept/Context) framework recommended by JBI to identify eligible studies as discussed below [14].

- Population: Tanzanian young population, as defined by the African Youth Charter for Sub-Saharan Africa [11], including ages from 15 to 35 years old.
- Concept: studies investigating obesity prevalence among the young population in Tanzania, the nutrition transition, and food policies.
- Context: only cohort, case-control, cross-section, observational studies and systematic reviews presenting data on the territories of the United Republic of Tanzania territory.

#### 2.2.2. Exclusion Criteria

Narrative reviews, conference proceedings, letters to editors, or short communications, publication of any other language than English, and studies including children under 15 years or adults above 35 years were excluded from the present scoping review.

### 2.3. Literature Search

**Figure 1:**
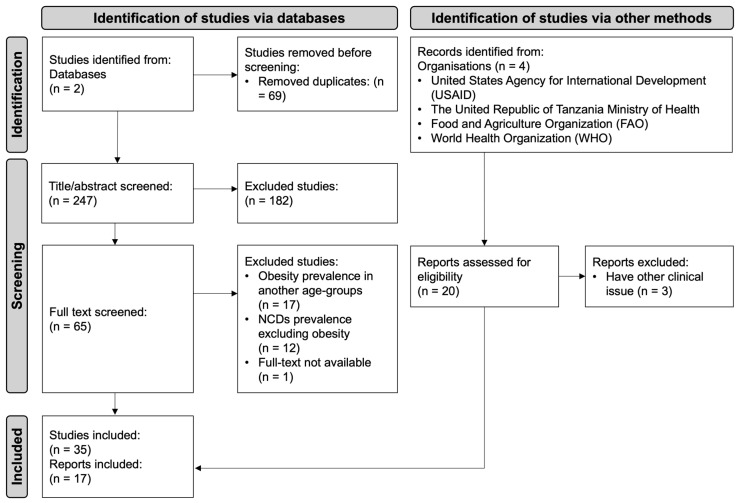
PRISMA flow chart describing the identification, screening, and selecting process of the included studies [15]. This work is licensed under CC BY 4.0. To view a copy of this license, visit https://creativecommons.org/licenses/by/4.0/ (accessed on 5 November 2024).

The search process yielded 164 articles on PubMed and 152 on Scopus on the 3rd of July 2025 (Addendum S1). Sixty-nine of these were found to be duplications and removed. The 247 remaining articles were screened for title and abstract. One hundred and eighty-two articles were removed for not meeting the eligibility criteria. The 65 remaining articles were screened for full text and 35 were found eligible to be included in this scoping review. Figure 1 below presents the PRISMA flow chart for the identification of the body of evidence to support the objective of this scoping review.

### 2.4. Data Extraction

Two researchers independently conducted the data extraction once studies for inclusion had been agreed on. In the instance of any discrepancies in data extracted, these were further assessed by a third independent researcher to ensure reaching a consensus on data quality.

## 3. Results

Among the 35 articles included in this scoping review, three major thematic areas emerged. These are presented in the following sections, organised according to the research questions guiding this study: (1) obesity prevalence and related determinants among youth in Tanzania; (2) evidence on the ongoing nutrition transition and associated lifestyle changes; and (3) national and local food-related policies aimed at addressing the rising prevalence of obesity among youth. The main features of the included studies are reported in Table 1, including author(s)/, year of publication, type of study, field of interest, study population characteristics, context, and main findings.

### 3.1. Obesity Prevalence and Related Determinants Among Youth in Tanzania

Our search identified nationwide reports [1,11,51,52,53] and cross-sectional studies [16,17,18,19,20,21,22,23,24,25,26,27,28,29,30,31,32,33,34,35,37] evaluating obesity in diverse age groups, ranging from young adolescents (10–15 years) to young adults (15–35 years). Although the African Youth Charter defines “youth” as individuals aged 15–35 years [11], epidemiological reports typically classify populations simply as children (≤18 years) or adults (≥19 years), resulting in an underrepresentation of youth-specific data. Moreover, certain subgroups, particularly women of reproductive age (15–49 years), receive disproportionate attention due to specific health risks, contributing to a knowledge gap regarding obesity among adolescents and young adults (15–35 years), and especially among young men [1,23,35].

According to the Global Nutrition Report [1], no East African country is on track to meet obesity reduction targets, and nine, including Tanzania, lack sufficient data to evaluate progress. Regional estimates show a clear upward trend: between 2000 and 2016, obesity prevalence in East African adults (≥18 years) increased from 5.2% to 10.1% in women and from 1.2% to 2.8% in men, while among children and adolescents (5–19 years), prevalence rose from 0.2% to 1.2% in boys and from 0.7% to 2.7% in girls [1].

Obesity is closely linked to other NCDs such as type 2 diabetes mellitus and hypertension, and studies highlighted that often both obesity and hypertension increase markedly with age [16,24,27]. However, young generations are not immune [21,26].

WHO estimates suggest that between 2000 and 2020, obesity increased more steeply among Tanzanian children and adolescents (5–19 years) than among adults, having quadrupled in both sexes, whereas prevalence tripled in adult men and increased 2.5-fold in adult women [1]. Nationally representative modelled estimates from the Global Burden of Disease 2019 indicate a doubling of adult obesity since 1990, with 5.4% of adult men and 12.8% of adult women (≥20 years) affected in 2019 [32]. Childhood and adolescent obesity (2–19 years) also doubled, reaching 5.3–5.4% in both sexes [32]. Earlier Demographic and Health Surveys further documented rising trends, with overweight (BMI ≥ 25 kg/m^2^) among women aged 15–49 years increasing substantially between 1996 and 2004/05 [18].

Although overall prevalence remains lower among children and adolescents compared with adults, stratified data reveal significant heterogeneity within the 5–19-year range. Data from the Global Health Observatory [51] show higher obesity prevalence in boys aged 5–9 years than in those aged 10–19 years (2.8% vs. 1.1%), with peak prevalence nearly three-fold higher (9.1% vs. 3.6%) [29,30]. In girls, prevalence is similar across the two age groups (1.7% vs. 1.8%), with peak values of 5% and 3.7%, respectively. Local studies further highlight these disparities: central obesity reached 22% among adolescents aged 14–19 years [33], while among urban university students (mean age 24–25 years) obesity prevalence reached 14% [31,37].

Gender disparities are consistently observed, with female adolescents and young women presenting significantly higher odds of overweight/obesity than males across both semi-rural and urban settings [16,17,19]. In the Babati District, obesity or overweight affected more females than male students (13.8% vs. 3%), and girls were nearly six times more likely to be affected (OR 5.6; 95% CI 1.97–15.72) [25]. Similar patterns were reported in Dar es Salaam, where females aged 15–25 and 26–35 years showed higher prevalence than males (26% vs. 12% and 55% vs. 42%, respectively) [20]. More recent urban data confirmed that female students were more likely to be overweight than male students (aRR 1.33; 95% CI 1.21–1.45), although adolescents aged 15–19 years constituted only a small proportion of the sample compared to younger adolescents (10–14 years) [36].

Beyond age and gender disparities, socioeconomic factors play a key role. A 2016 analysis of TDHS 2010 data found a higher prevalence of obesity among women of reproductive age in Zanzibar than in Mainland Tanzania (12.19% vs. 6.56%) [22]. Risk factors included higher wealth index, marriage, and being 30–49 years old, although these associations remained significant only in Mainland Tanzania, not in Zanzibar [22]. A large nationally representative study of 11,738 women [28] reported an overall overweight/obesity prevalence of 18.4% (obesity 10%), with higher values in the 25–34-year age group and in women with higher education, wealth, formal employment, alcohol consumption, and television viewing. A 2021 cross-sectional study in Dar es Salaam similarly found that older age (35–49 years), higher wealth status, informal employment, and marriage increased the risk of overweight/obesity, with higher sugar consumption associated with increased risk and fish and poultry consumption associated with lower risk [34]. Education level, household size, and physical activity showed no significant association [34].

A sub-analysis of global obesity trends (1980–2015) by age group and income level further suggests that obesity prevalence peaks earlier in middle-income countries (35–39 years) and later in low- and lower-middle-income countries (50–54 years), with increases beginning already in adolescence (15–19 years) [4]. These findings underscore the need for targeted interventions across adolescence, young adulthood, and middle age, adapted to the economic context of each country.

Finally, a large meta-analysis within the NCD Risk Factor Collaboration (NCD-RisC) [52] highlights a gender imbalance in Tanzanian obesity research, with more studies focusing on women than men (12 vs. 7) and more on girls than boys (5 vs. 2). This gap reinforces the need for improved gender- and age-stratified data to inform the development of effective, evidence-based prevention strategies.

Given these disparities, obesity epidemiology in Tanzania should be examined with greater stratification based on risk factors. Future research should explore socioeconomic determinants such as access to physical activity facilities, consumption of ready-to-eat foods, and nutrition education. Identifying specific subgroups at risk, particularly the youth, and considering regional disparities within Tanzania will be essential to designing effective interventions.

### 3.2. Evidence on the Ongoing Nutrition Transition and Associated Lifestyle Changes

Nationwide report [54,55,56] and studies [38,39,40,41,42,43,44,45,46,47,48] focusing on nutrition transition in youth were identified. Understanding changing dietary patterns is essential for designing effective nutrition interventions for NCD prevention. Economic development, urbanisation, and technological advancements have altered traditional lifestyles, including dietary habits. The global nutrition transition, driven by urbanisation, globalisation, and shifts in lifestyles, is transforming food habits in Africa [57], typically leading to reduced physical activity and increased intake of processed foods high in fats and sugars [58]. As described in the previous section, Tanzania is also undergoing this epidemiological and nutrition transition facing a rising of obesity and related NCDs. A recent study highlighted an association between obesity and several lifestyle factors such as habitual breakfast skipping, high soft drink consumption, regular fast-food intake, and low vegetable and fruit consumption [40]. Historically, Tanzanian diets were based on locally grown staples such as maize, millet, sorghum, starchy tubers (cassava and potatoes), and pulses (primarily kidney beans) [59]. These diets were rich in complex carbohydrates and fibre and low in saturated fats. Refined sugar, absent from traditional diets, became widespread in the 18th century to meet European demand and today is a major ingredient in confectionery and starchy snacks high in saturated fats, contributing to obesity and metabolic disorders [39]. In contemporary Tanzania, increased availability and affordability of processed, energy-dense foods, combined with rising disposable income, are accelerating the shift from heritage-style to Western-style dietary patterns, particularly in urban areas [38,60,61]. In particular, a rise in ultra-processed foods (UPF), such as such as sugar-sweetened beverages, cookies, or deep-fried foods, and the number of meals consumed away from home has emerged in recent years [44,59,62]. This transition is especially affecting younger generations, particularly during the transition to university, a critical period for young adults who are at increased risk of overweight and obesity [37].

This shift affects metabolic pathways associated with NCDs and promotes a pro-inflammatory state [42,48]. The rising prices of nutrient-rich foods and increased consumption of energy-dense snacks eaten away from home have been well documented [59]. This aspect persisted during the COVID-1 pandemic where economic and social vulnerability and reliance on markets (combined with lower agricultural production) were negatively associated with diet quality [46].

Although initially more pronounced in urban areas, the nutrition transition is now rapidly advancing in rural settings. In rural Tanzania, processed foods and meals consumed away from home account for around 70% of total food intake [62], and approximately 10% of rural diets are classified as ultra-processed [58]. These trends align with rising rates of overweight and obesity in rural communities. A recent study highlighted that the traditionally “plant-based pattern,” largely consisting of unprocessed and minimally processed foods, was inversely or not associated with overweight/obesity, whereas the “purchase pattern,” characterised by highly processed foods, showed a positive association [42,44]. Food crop diversity was positively associated with Prime Dietary Quality Score (PDQS) (*p* < 0.001); for women living close (<1.1 km) to markets, producing one additional food crop was associated with a 0.67 (95% CI, 0.22–1.12) increase in PDQS, compared with a 0.40 (95% CI, 0.24–0.57) increase for those living farther away [43].

The 2016 FAO Scale-N Nutrition Survey found that over 50% of daily carbohydrate (195 g/person) and fibre (27 g/person) intake in rural Tanzania comes from cereals [54]. Fruits and vegetables contribute only 7% of fibre, and pulses just 4%. Sweets and sugar account for 2.5% of carbohydrate intake, while most dietary fat comes from composite dishes, legume-based (33%), vegetable-based (20%), fish and seafood (13%), and meat (7%). Only 6.8% of surveyed women met the Minimum Dietary Diversity for Women (MDD-W), while 93.2% fell short. Similarly, another study found that only 34% of women achieved the MDD-W, with 33% consuming deep-fried foods [47].

Evidence consistently shows that dietary diversity is protective. A cross-sectional study from 2021 found that higher dietary diversity scores were associated with lower prevalence of abdominal obesity [41]. Similarly, Paulo et al. [45] reported that women in the highest PDQS quintile were significantly less likely to present overweight or obesity, with consumption of plant-based foods (such as green vegetables and pulses) offering protection, whereas high consumption of animal foods was a risk factor.

Micronutrient deficiencies, particularly iron and zinc deficiency among women of reproductive age, remain a major concern due to high reliance on cereals containing antinutritional factors such as phytates, which inhibit iron absorption [63]. For instance, in the Iringa region, the widespread practice of soaking maize before milling results in ultra-refined flour contributing to “empty calorie” intake, which is high in energy but low in essential nutrients such as niacin.

The 2022 Global Nutrition Report for Tanzania highlights substantial gaps in recommended intake of fruits (33%), vegetables (56%), and legumes (34%) [1]. Dietary differences between urban and rural areas are shaped by variations in wealth, education, food prices, and accessibility [63]. Similar patterns appear globally, as follows: across 28 LMICs, only 18% of individuals aged ≥ 15 years met the WHO recommendation of 400 g/day of fruits and vegetables, with mean intakes of 1.15 fruit and 2.46 vegetable servings per day [64].

Overall, there is a pressing need for comprehensive data on dietary patterns and food choice determinants across rural and urban settings, age groups, and genders in Tanzania. Understanding these dynamics is crucial for designing targeted, context-specific and equity-focused nutrition interventions that simultaneously address both ends of the malnutrition spectrum.

### 3.3. National and Local Food-Related Policies Aimed at Addressing the Rising Prevalence of Obesity Among Youth

There is scarce scientific evidence about obesity policy-making in Tanzania or the effectiveness of policies and initiatives in place to address obesity. Through our search we identified only two articles [49,50] and three main reports addressing obesity policy in Tanzania, mostly indirectly [65,66,67]; two studies addressed adolescents as a target group and how the school environment affects obesity and overweight incidence [50], while one study was a report of the annual NCDs week [68] where initiatives and policies addressing NCDs between 2020 and 2021 for the whole nation were discussed, and the other report was the latest National NCD Action Plan 2021–2026.

The NCDs week, held annually, focuses on (1) community awareness and participation encouragement through media as well as community-based preventive and advocacy activities; (2) physical activity promotion and sports festivals; (3) health education via school initiatives for secondary school adolescents; (4) health screening initiatives including screening for obesity; and (5) scientific conferences bringing together all stakeholders to discuss and recommend strategies for NCDs. However, screening during NCD week focused more on hypertension, diabetes, and alcohol intake and little attention was given to obesity prevalence, even during the screening initiatives [68].

The most recent National NCD Action Plan 2021–2026 in Tanzania identified obesity and overweight as a modifiable risk factor for NCDs, which they aim to address among all age groups, having set a target of 0% increase in obesity prevalence by 2020, which was not reached. The current plan emphasises prevention of obesity through nutrition and physical activity with interventions needed in the following: multisectoral coordination and governance, health promotion and awareness, integration of prevention in health services, environmental and policy interventions in food processing, dietary guidelines, and food advertising, resource mobilisation, and capacity building integrating surveillance, research, and monitoring. The challenges faced, however, to implement these strategies include resource constraints, lifestyle change complexity, competing health burdens (especially with infectious diseases), awareness and behaviour change, data and monitoring activities gaps, and cross-sectoral collaboration. One major challenge remains the fact that targets to reduce obesity prevalence are not specific neither for the population nor per age group. Monitoring of obesity prevalence is still segregated in adolescents (below 25 years old) and adults (25–64 years old) even though the African Union has created a separate age group to address the youth which includes 15–35-year-olds.

Tanzania also has the National NCDs strategy, national multisectoral nutrition action plan (NMNAP) II (2021–2026) [66], and the National Nutrition Social and Behavior Change Communication (SBCC) strategy [67]. In Tanzania, there are Regional and Council Multisectoral Steering Committees on Nutrition, also ward- and village-level committees on nutrition governance which meet quarterly to plan and discuss progress of different nutrition interventions. Terms of reference are created for these committees to support the implementation of NMNAP I&II [66]. Having such structures in place provide a strong foundation for policy actions in the prevention of obesity.

Despite the efforts in Tanzania to address NCDs through policies, there are gaps in their implementation and creating awareness, especially among target groups. The study by Nicholaus et al. [39] implemented in 31 boarding high schools in the Kilimanjaro region including 164 adolescents 16–19 years old (the majority of the adolescents were between 18 and 19 years old), revealed that diet in boarding schools was inadequate in that it was largely cereal–legume based and low in animal sources and fruits and vegetables. Consequently, mean energy, vitamin C, iron, calcium, and zinc intakes were below the Recommended Daily Allowance (RDA), while carbohydrates were well above the RDA for both sexes, protein intake was about 1.5 times the RDA for males and only slightly higher for females, and fat was lower than the RDA for males (58.3 g vs 73.4 g) and well above the RDA for females (89.0 g vs 73.4 g). Among these high school students, 3.2% were presented with obesity but 29% were overweight. These results present a lack of strategy or initiatives’ implementation for curbing obesity in schools through diet.

In a cross-sectional study, where knowledge and practices on obesity prevention were assessed among 253 secondary school adolescents in the Morogoro municipality, although over 80% of students reported being aware of the causes of obesity, only half knew its effects on hypertension and only 11.5% knew about its effects on diabetes development [50]. Although just over 50% of the students recognised physical activity as a preventive measure for obesity, only 27% mentioned vegetables and fruits to be healthy foods (same ref as above). Almost 50% mentioned high-fat foods as the unhealthiest foods, but about 21% of respondents did not know which were the unhealthy foods at all (same ref as above). Additionally, 75% of respondents reported doing exercise to prevent obesity and 60% said they participated in sports, while only 3% and 5% reduced high-fat foods and tried to follow a balanced diet, respectively (same ref). These results call for better communication of the effects of the diet on obesity, and better understanding of the perceptions of young adults throughout Tanzania.

## 4. Discussion

### 4.1. Recommendations for Policy Actions

There is scarce scientific evidence about obesity policy-making in Tanzania or effectiveness. While 10 years ago investment was still focused on communicable diseases in East Africa [69], the latest reports from large-scale programs, like the USAID Nutrition Program, suggests investments have shifted to nutrition-specific and nutrition-sensitive programs to mitigate the rapid rise in NCDs including obesity [55]. However, as analysed in this narrative review, data is still missing to effectively address diet-related NCDs in Tanzania, especially when prioritizing population subgroups and assessing the drivers of nutrition transition among age groups, especially for youth. In addition to the need of multisectoral collaboration between health, education, and food systems to address diet-related NCDs, national policies should address subgroups like youth considering their specific needs of a greater, more sustainable impact [7].

#### 4.1.1. Food Systems

Policies aimed at transforming food systems should focus on creating healthy food environments and ensuring access to sustainable, nutritious diets for all. To be effective, they must be context-specific and prioritise evidence-based interventions proven successful in similar settings. Several aspects of the food system can be addressed; here, we highlight key areas supported by research in Tanzania or comparable contexts.

Given Tanzania’s nutrition transition and the increasing presence of refined sugars in the diet despite limited data, estimates suggest an intake of approximately 5 g per person per day from sweets and sugars, with additional, unquantified intake from processed foods [54]. While Tanzania has implemented sugar and alcohol taxes, these primarily serve revenue generation rather than reducing consumption [70]. A study estimated that a 20% tax on sugar-sweetened beverages (SSBs) could lower energy intake by 18 kcal per person per day and reduce adult obesity prevalence by 6.6%, with higher effects in males (12.9%) than females (5.2%) [70]. A similar tax approach could be applied to alcohol to mitigate hypertension and obesity [71], though it remains unclear whether alcohol consumption patterns influence the obesity–age relationship in Tanzania [72].

In 2023, Tanzania introduced Food-Based Dietary Guidelines (FBDGs) in collaboration with FAO [56], following the launch of Zanzibar’s FBDGs. The Ministry of Health’s implementation plan emphasises multisectoral collaboration, particularly in public institutions. Effective policy actions should include (i) setting nutritional standards for meals in schools, hospitals, and public food services; (ii) strengthening food labelling policies to guide healthier consumer choices; (iii) regulating food imports and industry practices to align with FBDGs; (iv) utilizing FBDGs as educational tools in schools and hospitals. Successful implementation must be evidence-driven. Research has already identified key barriers such as cultural differences, limited nutrition knowledge, time constraints, and poverty [73]. Conceptual frameworks highlight effective models, such as school-feeding programs involving key stakeholders [74], while behavioural research can inform policy-makers on the drivers of food choices across different age groups [75].

Finally, empowering key community stakeholders, especially women, is crucial for food system transformation. Women’s empowerment has been linked to improved dietary diversity and better nutrition outcomes, with autonomy, decision-making in food production, and public engagement being key enablers [76]. Policies should support and strengthen their role to drive sustainable change.

#### 4.1.2. Physical Activity Promotion

Reduced physical activity, that accompanies the nutritional transition observed in Tanzania, has been described as one of the main risk factors of obesity [53,77]. It is therefore essential to create an enabling environment for physical activity in different contexts including neighbourhood common areas, school grounds, work sites, and other areas where people spend their free time.

Investments in infrastructure have rapidly increased in Tanzania [78], with significant improvements in electrification of different areas including rural ones, and public transportation connecting rural to urban areas [79]. The infrastructure investment has, in fact, reached 10.6% gross domestic product (GDP) [79]. However, such infrastructure developments should be accompanied by plans to incorporate an enabling environment for physical activity, along with campaigns to specifically promote physical activities among target age groups. Furthermore, to target the needs of age subgroups, health needs assessments should be carried out in different contexts such as school grounds, work sites, and community centres, to better inform investments and policy actions in promoting physical activity.

In Tanzania, an infrastructure policy which was implemented back in the 1970s and 1980s, which included providing large greenfield areas in growing neighbourhoods for people to build their own homes, was more recently analysed and found to have been a policy action worth revisiting [78]. These so-called “Sites and Services” projects provided greenfield areas where people built their own houses and resulted in higher quality housing and better neighbourhoods, preventing the formation of slums. This program is cheaper for the government than building homes and renders more durable and sustainable housing in greenfield areas with less waste. Modern policy could leverage lessons learnt from such projects and further develop them, in collaboration with the residents and supported by urban planning experts, to design and include football areas, outdoor gyms, and playgrounds to promote physical activity.

School environments have been found to be adequate to promote physical activity; however, the quality and use of playgrounds at schools have not been up to standards [80]. Leveraging existing infrastructure and integrating policy-mandated interventions [81] may be a more effective approach to promote physical activity in Tanzania, targeting WHO recommendations [82].

#### 4.1.3. Public Education Campaigns

Public education campaigns play a crucial role in shaping health behaviours in Tanzania, addressing nutrition and obesity prevention, reproductive health, and tobacco control. Large-scale initiatives like United Nations International Children’s Emergency (UNICEF) school enrolment campaign, which aims to reintegrate over 100,000 children, highlights the interconnectedness of education and public health. Ensuring children remain in school not only enhances literacy but also provides opportunities for structured nutrition and health education, which are critical for fostering lifelong healthy habits [83]. Nutrition education has been a central integration into school curricula as a strategy for obesity prevention and long-term dietary improvements [84]. Initiatives such as the nutrition-sensitive education programs promoted by international organisations like UNICEF and Catholic Relief Services (CRS) have focused on improving maternal and child nutrition through targeted interventions, particularly in rural areas where malnutrition remains a major concern [83,84]. However, a situation analysis conducted in Dodoma highlighted that, while schools provide a valuable setting for nutrition education and meal programs, limitations such as inconsistent food supply, lack of trained staff, and insufficient government funding hinder their effectiveness [81].

A notable initiative in health and nutrition is the project led by the International Development Law Organization (IDLO), which promotes healthy diets and physical activity through policy advocacy and multisectoral collaboration [85]. One of the strengths of this initiative is its policy-focused approach, ensuring that healthy lifestyle promotion is embedded within Tanzania’s public health strategies. However, challenges remain in translating advocacy into action, particularly in rural areas where access to nutritious food and safe spaces for physical activity is limited [85].

Another key area of public education campaigns is reproductive health, including prenatal and pregnancy nutrition education. In Tanzania, the increasing prevalence of obesity among women of reproductive age has been associated with obstetric complications that can negatively impact neonatal health [86]. A high maternal BMI is linked to higher birth weight, increasing the child’s risk of obesity. Exclusive breastfeeding for the first six months of life is crucial for infant health, reducing the risk of future obesity [87]; however, in Tanzania, the rate of exclusive breastfeeding is still low at 57.8% [1]. Targeted interventions, including young girls, to improve knowledge about healthy diets during pregnancy, breastfeeding practices, and the importance of essential micronutrients, can help reduce both maternal and infant mortality and the risk of childhood obesity. Campaigns like those led by Tanzania Network of Community Health Workers (TANCO) have played a vital role in spreading awareness, particularly among adolescents and young women, emphasizing the importance of early nutrition interventions [88].

Tanzania has implemented anti-tobacco campaigns, recognizing the impact of tobacco consumption on NCDs. Government policies have focused on discouraging smoking through mass media campaigns and public health messaging. However, the effectiveness of tobacco control policies in Tanzania remains limited due to gaps in enforcement, particularly regarding public smoking and advertising [89]. Additionally, outdated data report staggering prevalences of cigarette smoking among the youth, especially in schools (4.8% of students) [90], which calls for implementation of tobacco control among underaged adolescents. While students seem to acknowledge the risks of passive smoking, the rate of exposure at home was at 17.3% in 2016 [90], highlighting the need to hold education campaigns for older adults too.

## 5. Strengths and Limitations

This scoping review has several strengths. To our knowledge, it is the first review to systematically map and integrate evidence on obesity among youth aged 15–35 years in Tanzania, bringing together three interconnected domains that are rarely examined in combination: epidemiological patterns, nutrition and lifestyle transitions, and policy responses. The use of the PCC framework and a structured data extraction process allowed for a comprehensive mapping of available studies, while the inclusion of both peer-reviewed literature and grey literature broadened the scope and contextual relevance of the findings.

However, several limitations must be acknowledged. First, only 35 studies met the inclusion criteria, mostly regarding obesity prevalence, reflecting the overall scarcity of research specifically focused on youth in Tanzania. Many studies reported wide and heterogeneous age ranges, which limited the possibility of isolating youth-specific data and often prevented meaningful subgroup analyses. In addition, substantial gaps in contextual information, such as socioeconomic indicators, regional variations, and environmental determinants, restricted the depth of interpretation. Evidence on nutrition transition and lifestyle changes was similarly fragmented, with few studies providing detailed or comparable measures related to dietary patterns, physical activity, or food environments.

Limitations were also observed in the policy-focused literature, where available documents were broad in scope, insufficiently detailed, and rarely tailored to the youth population. As a result, drawing firm conclusions on policy implementation, effectiveness, or youth-specific relevance was challenging. Finally, although efforts were made to capture the grey literature, relevant documents may not have been identified due to limited public access or incomplete indexing.

Despite these limitations, this review offers a consolidated overview of the current evidence base and highlights clear gaps that can inform future research, surveillance systems, and the development of more targeted youth-centered policies in Tanzania.

## 6. Conclusions

This scoping review mapped the existing evidence on youth obesity in Tanzania, with a focus on epidemiological data gaps, nutrition and lifestyle transitions, and the policy landscape addressing this issue. Our findings clearly show that evidence specific to youth aged 15–35 years remains limited, inconsistently reported, and often embedded within broader adult age groups, hindering a clear understanding of obesity patterns within this population. The review also highlights fragmented and incomplete documentation of the ongoing nutrition transition and its implications for young people, as well as policy frameworks that, while active, lack youth-specific objectives and measurable targets. By bringing together these three interconnected domains, this review contributes a consolidated picture of where the evidence currently stands and where critical gaps persist. Addressing these gaps will require strengthening age-disaggregated epidemiological surveillance, generating more context-specific research on dietary and lifestyle behaviours among youth, and developing policy actions that explicitly target this age group. Future studies should also evaluate the long-term sustainability, cultural acceptability, and behavioural impact of interventions designed for young people, as such evidence is almost entirely absent.

Overall, this review underscores the need for a more youth-centered approach in both research and policy. Enhancing multisectoral coordination, ensuring culturally adapted strategies, and reinforcing implementation capacity will be essential to support Tanzania’s efforts to curb the rising burden of youth obesity and related non-communicable diseases.

## Figures and Tables

**Table 1:** Main features of the 35 included studies.

Field of Interest	First Author, Year	Type of Study	Population (*n*, Age)	Context (Rural/Urban)	Summary of Findings
Obesity prevalence	Nyaruhucha, C. N. M. et al., 2003 [16]	Cross-sectional study	*n* = 140 Sex not available Age: Age 19–50 years: 71.43% Age 14–18 years: 28.57%	Urban	The prevalence of obesity among the sampled subjects in Morogoro Municipality was 25%, whereby 15.7% had a body mass index (BMI) of between 25 and 30, and 9.3% had a BMI of more than 30. Age and occupation of all the subjects, together with marital status of adult subjects, were significantly related with obesity status. Prevalence of obesity increased with the increased age, whereby subjects of 41–50 years had the highest rate (45.4%). Employed subjects had higher rate of obesity (22.2%) than pupils or students. Similarly, married adults had higher rate of obesity (27.8%) than the single ones (4.7%). Unlike the old age group (41–50 years), 70% of the youngest subjects were not aware about the harmful effects of obesity.
	Villamor, E. et al., 2006 [17]	Cross-sectional study	*n* = 73,689 women Age: 14–52 years	Urban	The prevalence of obesity rose steadily and progressively from 3.6% in 1995 to 9.1% in 2004 [adjusted prevalence ratio (PR): 1.97; 95% CI: 1.66, 2.33; P for trend for year 0.0001]. Underweight showed only a modest decline from 3.3% in 1995 to 2.6% in 2004 (adjusted PR: 0.91; 95% CI: 0.75, 1.10; P for trend for year 0.003), whereas no change was observed in the prevalence of wasting. In the most recent years (2003 and 2004), obesity was positively associated with age, parity, and socioeconomic status and inversely with HIV infection.
	Jones-Smith, J.C et al., 2011 [18]	Cross-sectional survey	*n* = 17,021 women Age range: 15–49 years	Urban/Rural	In just eight years, overweight has increased mainly in the wealthiest quintiles (from 30.3% to 42.8%), but also in the middle quintiles. In the poorest quintile, the increase was minimal. The SII (slope index of inequality) went from −24.9 in 1996 to −39.7 in 2004, and inequality increased sharply.
	Shayo, Grace A. et al., 2011 [19]	Cross-sectional study	*n* = 1249, 65.2% females Age groups: 18–24 years: 26.4%; 25–34 years: 33.1%; 35–44 years: 20.1%; 45–54 years: 11.5%; 55+ years: 8.9%	Urban	The overall prevalence of obesity was 19.2% (240/1249). However, obesity was significantly more prevalent in women (24.7%) than men (9%), *p* < 0.001.
	Malliga, E. et al., 2013 [20]	Cross-sectional study	*n* = 44,120 primary school adolescent Age range: 10–14: 90.4%; 15–19: 9.6%	Urban	The prevalence of anaemia was 34.1%, while stunting and overweight had a prevalence of 32% and 4.2%, respectively. Approximately 41.7%, 13.5%, and 0.3% had single, double, and triple burden malnutrition-related conditions, respectively. Females were found to have a higher risk of being overweight compared with males
	Mushengezi, B. et al., 2014 [21]	Cross-sectional study	*n* = 582 adolescents, 52.1% boys Age: (mean, SD) 16.5±1.8 years,	Urban	The proportion of adolescents with overfat or obesity was 22.2%. Systolic, diastolic and combined hypertension was present in 17.5%, 5.5%, and 4.0% respectively. In the total population mean body fat percent correlated positively with diastolic blood pressure and mean arterial pressure
	Paul, E. et al., 2016 [22]	Cross-sectional survey	*n* = 9131 women Age: 15–19 years: 22.7% 20–29 years: 32.7% 30–39 years: 25.9% 40–49 years: 18.7%	Urban/Rural	About 7.92% of the Tanzanian women of reproductive age were obese, 15% were overweight, and 11.5% were underweight. Women from Mainland Tanzania (6.56%) were significantly less likely (AOR = 0.66, 95% CI: 0.53–0.82) to be affected by obesity as compared to women from Zanzibar (12.19%).
	Amugsi, D.A. et al., 2017 [23]	Cross-sectional survey	*n* = 5115 non-pregnant women Age: 15–49 years.	Urban	Overweight prevalence was 14.1% in 1991, 20.5% in 1996, 18.9% in 2004, and 21% in 2009. Obesity prevalence was 3.6% in 1991, 7.8% in 1996, 9.7% in 2004, and 11.8% in 2009.
	Msemo, O.A. et al., 2018 [24]	Cross-sectional study	*n* = 2629 women Age, median (range): 28.0 (18–40) years.	Rural	The age-standardised prevalences of pre-hypertension and hypertension were 37.2 (95% CI 34.0–40.6) and 8.5% (95% CI 6.7–10.8), respectively. The prevalence of obesity was 5.25 among the overall sample; in particular, 32.6% in normotensive women, 51.5% in pre-hypertensive women, and 15.9 in hypertensive women. In multivariate analysis, increasing age, obesity, and haemoglobin levels were significantly associated with pre-hypertension and hypertension.
	Tluway, F.D. et al., 2018 [25]	Cross-sectional survey	*n* = 619, 42.8% males Age (mean, SD): 16.7 ± 1.68 years	Semi-rural	The overall prevalence of overweight and obesity was 9.2% with more girls being overweight and obese than boys (*p* < 0.0001).
	Nsanya, Mussa K. et al., 2019 [26]	Cross-sectional study	Tanzania *n* = 891, 58% males Age (mean, SD): 19.2 (±3.1)	Urban	Overweight/obesity prevalence: 12% The overall prevalence of high blood pressure was 40%. The prevalence of pre-hypertension was 29% and that of hypertension was 11%. High blood pressure was independently associated with obesity, male sex, and among males aged above 20 years. Consumption of fruits/vegetables was associated with decreased odds for high blood pressure (aOR = 0.7, 95% CI: 0.50–0.98).
	Nyangasa, M.A. et al., 2019 [27]	Cross-sectional study	*n* = 470, 47.4% males Age (mean, SD): 29 ± 18 years	Urban/rural	The proportion of overweight/obese individuals was 26.4%. Obesity and hypertension significantly increased with age and were most prevalent in participants aged 45 years and above.
	Ahmed, Kedir Y. et al., 2020 [28]	Cross-sectional study	*n* = 11,738 women Age: 15–24 years: 40.0% 25–34 years: 28.2% 35–49 years: 31.8%	Urban/Rural	Among the overall sample, overweight and obesity prevalence was 18.4% and 10%, respectively. Among 15–24 years, overweight and obesity prevalence was 12% and 2.8%, respectively. Among 25–34 years, overweight and obesity prevalence was 21.9% and 11.9%, respectively. Reproductive-age women who attained secondary or higher education (RRR = 1.48; 95% CI: 1.11, 1.96), those who resided in wealthier households (RRR = 2.31; 95% CI: 1.78, 3.03), and those who watched television (RRR = 1.26; 95% CI: 1.06, 1.50) were more likely to be overweight. The risk of experiencing obesity was higher among reproductive-age women who attained secondary or higher education (RRR = 1.79; 95% CI: 1.23, 2.61), those who were formally employed (RRR = 1.50; 95% CI: 1.14, 1.98), those who resided in wealthier households (RRR = 4.77; 95% CI: 3.03, 7.50), and those who used alcohol (RRR = 1.43; 95% CI: 1.12, 1.82) and/or watched the television (RRR = 1.70; 95% CI: 1.35, 2.13).
	Darling, A.M. et al., 2020 [29]	Cross-sectional study	Tanzania urban: *n* = 743 Age range 10–14 years: 66.7% 15–19 years: 33.3% Tanzania rural: *n* = 825 10–14 years: 32.3% 15–19 years: 67.7%	Urban/rural	The prevalence of overweight in urban Tanzania was 5.1%. The prevalence ratio of overweight in rural Tanzania was 1.39 (95% CI, 0.80, 2.42).
	Ismail, A. et al., 2020 [30]	Cross-sectional survey	*n* = 1226, 44.7% males Age (mean, SD): 13.7 (2.25)	Rural	Overweight and obesity affected 5.23% of participants. Girls had higher HAZs (b: 0.46, 95% CI 0.33, 0.59, *p* < 0.0001) and body mass index (BMI)-for-age-z-scores (BAZs) (b: 0.20, 95% CI 0.05, 0.35, *p* = 0.0098) than boys. Age was inversely associated with height-for-age-z-scores (HAZs) (b: 0.13, 95% CI 0.17, 0.08, *p* < 0.0001) and BAZs (b: 0.05, 95% CI 0.10, 0.004, *p* = 0.0327).
	Tengia-Kessy, A. et al., 2020 [31]	Cross-sectional study	*n* = 400 secondary school girls Age (mean, SD): 15.1 ± 1.5 years	Urban	The proportion of adolescents with excess body weight (BMI > +1SD) was 23%. The majority (63%) reported unhealthy dietary habits while half (51.5%) of them had a moderate level of knowledge on healthy eating.
	Gona, P.N. et al., 2021 [32]	Epidemiological analysis	GBD 2019 population-level estimates for Tanzania (no primary sample; modelled for entire national population, all ages)	Urban/rural	The age-standardised prevalence of obesity (BMI ≥ 30 kg/m^2^) in Tanzania showed a marked increase between 1990 and 2019. In adults aged 20 years and older, obesity prevalence in 2019 was estimated at 5.4% among males (95% uncertainty interval 4.4–6.5%) and 12.8% among females (95% UI 11.2–14.6%). This represents more than a doubling of obesity over the 29-year period for both sexes, with the rise being particularly pronounced in women. Among children and adolescents aged 2–19 years, obesity prevalence in 2019 reached 5.4% in boys (95% UI 4.4–6.5%) and 5.3% in girls (95% UI 4.3–6.5%), again roughly doubling from the levels observed in 1990.
	Lwabukuna, W.C. et al., 2021 [33]	Cross-sectional study	*n* = 217, 32% males Age: Young adolescents (14–17 years): *n* = 162 (75%) Elder adolescents (18–19 years): *n* = 55 (25%)	Urban	The prevalence of full-blown metabolic syndrome was 1.4% (3). Overall, the clinical markers included dyslipidaemia 30% (64), central obesity 22% (48), hyperglycaemia 13% (29) and hypertension 2% (4). Prevalence of central obesity was 26% (42) among young adolescents and 11% (6) among elderly adolescents and the difference was significant (*p* value = 0.02).
	Mosha, D. et al., 2021 [34]	Cross-sectional survey	*n* = 1004 women Age (mean, SD): 30.2 (±8.1) years.	Urban	Prevalence of overweight and obesity was high (50.4%), and underweight was 8.6%. The risk of overweight/obesity was higher among older women (35–49 vs 15–24 years: PR 1.59; 95% CI: 1.30–1.95); women of higher wealth status (PR 1.24; 95% CI: 1.07–1.43); and informally employed and married women. Attaining moderate to high physical activity (≥600 MET) was inversely associated with overweight/obesity (PR 0.79; 95% CI: 0.63–0.99). Dietary sugar intake (PR 1.27; 95% CI: 1.03–1.58) was associated with increased risk, and fish and poultry consumption (PR 0.78; 95% CI: 0.61–0.99) with lower risk of overweight/obesity.
	Gibore, N.S. et al., 2023 [35]	Cross-sectional study	*n* = 749, 42.1% males Age (mean, SD): 47.6 ± 14.3 years	Urban	Overall, 63.5% (33.3% overweight and 29.9% obese) were overweight or obese, 4.5% were diabetic, and 43.4% were hypertensive. Only 35.4% of participants had adequate knowledge of CVDs risk factors.
	Mchau, G. et al., 2024 [36]	Cross-sectional study	*n* = 44,120 primary school adolescents Age range: 10–14: 90.4% 15–19: 9.6%	Urban	The prevalence of anaemia was 34.1%, while stunting and overweight had a prevalence of 32% and 4.2%, respectively. Approximately 41.7%, 13.5%, and 0.3% had single, double, and triple burden malnutrition-related conditions, respectively. Females were found to have a higher risk of being overweight compared with males
	Mgetta, N.J. et al., 2024 [37]	Cross-sectional study	*n* = 247, 53% males Age range: 24–25 years: 62.8% >25 years: 37.2%	Urban	Overweight prevalence was 21.8%, while obesity prevalence was 14%. University students are a vulnerable group in developing obesity/overweight due to the transitional stage. Being overweight and obese was associated with being female, increased age, and being married. High dietary diversity was also linked with abdominal obesity.
Nutrition transition	Muhihi, A. et al., 2011 [38]	Cross-sectional survey	*n* = 97 men Age (mean, SD): 31.6 ± 6.4 years.	Urban	Obesity prevalence: 4.1%. More than half (53.6%) of the participants had energy expenditure of ≥4000 kcal/week. Physical activity energy expenditure was high in this population and was inversely correlated with CVD risk factors.
	Nicholaus, C. et al., 2020 [39]	Cross-sectional study	*n* = 164, 31.7% males Age: mean (SD) 18.3(±0.7)	Urban/Rural	Mean intake of energy, vitamin C, iron, calcium, and zinc was 1392 kcal, 24.8 mg, 9.2 mg, 134.5 mg, and 4.3 mg, respectively, which were below the Recommended Daily Allowance. Average carbohydrate, fat, and protein intake of 471.9 g, 73.7 g, and 80.7 g, respectively, were slightly higher than the Recommended Daily Allowance in both sexes. Males had a significantly higher intake of protein and carbohydrates (*p* < 0.001). Females had a significantly (*p* < 0.001) high intake of fat compared to male adolescents. Overall, 23.1% of the adolescents were anaemic, 25% were overweight, and 6.1% were obese.
	Pallangyo, P. et al., 2020 [40]	Cross-sectional study	*n* = 6691, 54.2% males Age: 43.1 years (IQR: 18–95)	Urban	Obesity prevalence: 32.4%. Overweight: 34.8%. Factors that significantly associated with obesity were age ≥ 40, being female, a current working status, habitual breakfast skipping, poor water intake, high soft drink consumption, regular fast-food intake, low vegetable and fruit consumption, alcohol consumption, and hypertension.
	Khamis, A.G. et al., 2021 [41]	Cross-sectional study	*n* = 510, 52.7% females Age median (IQR): 36 (52–25)	Urban/rural	The prevalence of general obesity based on BMI was 20.2% (95%CI; 16.9–23.9), abdominal obesity based on WHR was 37.8% (95%CI; 33.7–42.1), and WC was 29.1% (95%CI; 25.2–33.1). More than half (54.3%) of the participants consumed an adequate dietary diversity (DDS > 4).
	Keding G.B. et al., 2021 [42]	Cross-sectional survey	*n* = 252 women Age (years): Range: 16–45 Mean ± SD: 33.3 ± 6.9	Rural	The five dietary patterns were “traditional- coast,” characterised by fruits, nuts, starchy plants, and fish; “traditional-inland,” characterised by cereals, oils and fats, and vegetables; “purchase,” characterised by bread and cakes (usually fried in oil), sugar, and black tea; “pulses,” characterised mainly by pulses, with few or no vegetables; and “animal products,” characterised by a high consumption of meat, eggs, and/or milk. Significant positive associations were found, among others, between the purchase pattern and BMI (ρ = 0.192, *p* = 0.005) and between the animal products pattern and wealth (ρ = 0.168, *p* = 0.002).
	Madzorera I., et al., 2021 [43]	Cross-sectional study	*n* = 868 women Age (mean, SD): 31.5 (± 7.7) year	Rural	There was a high prevalence of maternal overweight (24.3%) and obesity (13.1%). Food crop diversity was positively associated with prime diet quality score (*p* < 0.001). For women living close (<1.1 km) to markets, producing 1 additional food crop was associated with a 0.67 (95% CI, 0.22–1.12) increase in prime diet quality score, versus a 0.40 (95% CI, 0.24–0.57) increase for women living farther away.
	Sarfo, J. et al., 2021 [44]	Cross-sectional and longitudinal study	*n* = 292 women Age (mean, SD): 32.24 ± 8.55	Rural	In Tanzania, the overweight/obesity rate was 42%. Several patterns were identified, yet a “plant-based pattern” largely characterised by unprocessed and minimally processed foods and a “purchase pattern” mainly distinguished by highly processed foods were dominant. The “plant-based pattern” was inversely or not associated with overweight/obesity, while the “purchase pattern” had a positive association or no association.
	Paulo, H.A. et al., 2022 [45]	Cross-sectional study	*n* = 1004 non-pregnant women Age: 30.2 (±8.1) years	Urban	Prevalence: 27.8% were overweight and 22.6% were obese. All 1004 women in the study consumed starchy staple foods. Of all the women studied, 10.5%, 1.7%, and 3.8% consumed vitamin A rich dark green vegetables, nuts and seeds, and beans and peas, respectively. Compared with women in the lowest quintile of Prime Dietary Quality Score (PDQS), those who were in the highest quintile were significantly less likely to be overweight or obese (Adjusted Prevalence Ratio (APR) = 0.76, 95% CI: 0.62, 0.89) (F for trend = 0.029). Risk factors included the highest consumption of animal foods (APR = 2.81, 95% CI: 1.51–3.51) and fast food (APR = 2.57, 95% CI: 1.24–4.34).
	Ismail, A. et al., 2023 [46]	Cross-sectional study	*n* = 654, 57.8% Age (mean, SD): 42.4 ± 12.5 years	Rural/Urban	Higher food prices and lower diet quality persisted during the COVID-19 pandemic. Economic and social vulnerability and reliance on markets (and lower agriculture production) were negatively associated with diet quality. Although recovery was evident, consumption of healthy diets remained low.
	Mwanri, A.W. et al., 2025 [47]	Cross-sectional study	*n* = 512 women of reproductive age Age range: 15–25 years: 55% 26–35 years: 32% >35 years: 13%	Rural	About 42% of the women had no formal education and about one in three women owned a mobile phone. About 70% consumed vegetables while 33% consumed deep-fried foods. Only 34% of the women met the minimum diet diversity (MDD-W) of five or more food groups. The mean NCD-protect score was 2.8 ± 1.4 and the NCD-risk score was 0.77 ± 0.97.
	Temba, G.S. et al., 2025 [48]	Randomised controlled trial	*n* = 77 men Age: 25.6 years (IQR: 21–27.2)	Rural/Urban	The switch from heritage-style to Western-style diet affected different metabolic pathways associated with non-communicable diseases and promoted a pro-inflammatory state with impaired whole-blood cytokine responses to microbial stimulation.
Policies	Njiro, B.J. et al., 2023 [49]	Report	*n* = 5528	Urban/Rural	In 2021, among a total of 2030 individuals screened for hypertension, 950 (46%) had high blood pressure; of these, about a third (31%) were newly diagnosed, and 15% were known hypertensive, either controlled on medications or uncontrolled. During the same period, we screened a total of 2026 individuals for diabetes; 10.1% of these had raised blood glucose, with newly diagnosed individuals comprising 3% of these. A total of 1472 people were also screened for obesity and about one third (29.6%) of the people did not meet the WHO-recommended 150 min of physical activity per week. Moreover, 35% of individuals reported not taking fruits and vegetables for five or more days per week. About 16% of females and 27% of males screened reported alcohol intake; with 5% of males reporting daily alcohol intake.
	Msollo, S.S. et al., 2025 [50]	Cross-sectional study	*n* = 253, 49% males Age: secondary school (not specified)	Urban	Only 20.2% (*n* = 51) and 43.5% (*n* = 110) reported consuming fruits and vegetables 7 days a week, respectively. Most of the participants (82.2%, *n* = 208) were aware of the causes of overweight and obesity, and increasing physical activity (51.8%, *n* = 131) was the most cited preventive measure. Being in a higher level of study was significantly associated with increased knowledge and practices on prevention of overweight and obesity.

## Data Availability

Data is contained within the article or Supplementary Material.

## Supplementary Materials

The following supporting information can be downloaded at https://www.mdpi.com/article/10.3390/nu18010061/s1; Addendum S1: Literature search on Pubmed and Scopus following the research questions.

## Abbreviations

The following abbreviations are used in this manuscript:

BMI	Body mass index
BP	Blood pressure
CRS	Catholic Relief Services
FAO	Food and Agriculture Organization
FBDGs	Food-Based Dietary Guidelines
GDP	Gross domestic product
JBI	Joanna Briggs Institute
IDLO	International Development Law Organization
LMICs	Low-middle income countries
MDD-W	Minimum Dietary Diversity for Women
MDG	Millenium Development Goals
NCDs	Non-communicable diseases
NCD-RisC	NCD Risk Factor Collaboration
NEPAD	New Partnership for Africa’s Development
NMNAP	National multisectoral nutrition action plan
PCC	Population / Concept / Context
PDQS	Prime Dietary Quality Score (PDQS)
PRISMA	Systematic Reviews and Meta-Analyses
PROSPERO	Prospective Register of Systematic Reviews
RDA	Recommended Daily Allowance
SBCC	Social and Behavior Change Communication strategy
TANCO	Tanzania Network of Community Health Workers
TDHS	Tanzania Demographic and Health Survey
UNICEF	United Nations International Children’s Emergency Fund
UPF	Ultra processed foods
WHO	World Health Organization
