# Supplementary material for: Obesity in Tanzanian Youth (15–35 Years): From Nutrition Transition to Policy Action—A Scoping Review
Source: Nutrients. 2025 Dec 24;18(1):61. doi: 10.3390/nu18010061 (PMC12787505; doi:10.3390/nu18010061)
Supplement: Supplementary file 1 [file nutrients-18-00061-s001.zip › Addendum S1.pdf]

nutrients

MDPI

Supplementary Materials for

Youth Obesity in Tanzania: From Nutrition Transition to Policy Action—A Scoping Review

Addendum S1. Literature research

1. What are the gaps in obesity prevalence data among the youth in Tanzania?

Pubmed	Scopus
(obesity[MeSH Terms] OR overweight[MeSH Terms] OR obesity[Title/Abstract] OR overweight[Title/Abstract]) AND (prevalence[MeSH Terms] OR incidence[MeSH Terms] OR prevalence[Title/Abstract] OR incidence[Title/Abstract] OR frequency[Title/Abstract] OR epidemiology[Subheading] OR epidemiology[Title/Abstract] OR burden[Title/Abstract] OR rate[Title/Abstract] OR rates[Title/Abstract]) AND (Adolescent[MeSH Terms] OR "Young Adult"[MeSH Terms] OR Students[MeSH Terms] OR youth[Title/Abstract] OR "young people"[Title/Abstract] OR "young adults"[Title/Abstract] OR adolescents[Title/Abstract] OR teenagers[Title/Abstract] OR "university students"[Title/Abstract] OR "college students"[Title/Abstract] OR "secondary school students"[Title/Abstract]) AND (Tanzania[MeSH Terms] OR Tanzania[Title/Abstract]) AND ("2000/01/01"[Date - Publication] : "3000"[Date - Publication]) NOT (review[Publication Type] NOT systematic[sb])	(TITLE-ABS-KEY (obesity) OR TITLE-ABS-KEY (overweight)) AND (TITLE-ABS-KEY (prevalence) OR TITLE-ABS-KEY (incidence) OR TITLE-ABS-KEY (frequency) OR TITLE-ABS-KEY (epidemiology) OR TITLE-ABS-KEY (burden) OR TITLE-ABS-KEY (rate) OR TITLE-ABS-KEY (rates)) AND (TITLE-ABS-KEY (youth) OR TITLE-ABS-KEY ("young people") OR TITLE-ABS-KEY ("young adults") OR TITLE-ABS-KEY (adolescents) OR TITLE-ABS-KEY (teenagers) OR TITLE-ABS-KEY ("university students") OR TITLE-ABS-KEY ("college students") OR TITLE-ABS-KEY ("secondary school students") OR TITLE-ABS-KEY (students)) AND (TITLE-ABS-KEY (tanzania)) AND (PUBYEAR > 1999) AND NOT (DOCTYPE (re))
Articles found at 03/07/25= 115	Articles found at 03/07/25= 38

2. What are the gaps in the evidence on nutrition transition based on dietary changes reported among the youth in Tanzania?

Pubmed	Scopus
("nutrition transition"[Title/Abstract] OR "dietary transition"[Title/Abstract] OR "nutrition shift"[Title/Abstract] OR "dietary patterns"[Title/Abstract] OR "nutrition patterns"[Title/Abstract] OR "changing diet"[Title/Abstract] OR "dietary change"[Title/Abstract] OR "food systems"[Title/Abstract] OR "westernization of diet"[Title/Abstract] OR "urban diet"[Title/Abstract] OR "processed food"[Title/Abstract] OR "ultra-processed food"[Title/Abstract] OR "nutrition transition"[MeSH Terms]) AND (Tanzania[Title/Abstract] OR Tanzania[MeSH Terms]) AND (Adolescent[MeSH Terms] OR "Young Adult"[MeSH Terms] OR Students[MeSH Terms] OR youth[Title/Abstract] OR "young people"[Title/Abstract] OR "young adults"[Title/Abstract] OR adolescents[Title/Abstract] OR teenagers[Title/Abstract] OR "university students"[Title/Abstract] OR "college students"[Title/Abstract] OR "secondary school students"[Title/Abstract]) AND ("2000/01/01"[Date - Publication] :	(TITLE-ABS-KEY ("nutrition transition") OR TITLE-ABS-KEY ("dietary transition") OR TITLE-ABS-KEY ("nutrition shift") OR TITLE-ABS-KEY ("dietary patterns") OR TITLE-ABS-KEY ("nutrition patterns") OR TITLE-ABS-KEY ("changing diet") OR TITLE-ABS-KEY ("dietary change") OR TITLE-ABS-KEY ("food systems") OR TITLE-ABS-KEY ("westernization of diet") OR TITLE-ABS-KEY ("urban diet") OR TITLE-ABS-KEY ("processed food") OR TITLE-ABS-KEY ("ultra-processed food")) AND (TITLE-ABS-KEY (tanzania)) AND (TITLE-ABS-KEY (youth) OR TITLE-ABS-KEY ("young people") OR TITLE-ABS-KEY ("young adults") OR TITLE-ABS-KEY (adolescents) OR TITLE-ABS-KEY (teenagers) OR TITLE-ABS-KEY ("university students") OR TITLE-ABS-KEY ("college students") OR TITLE-ABS-KEY ("secondary school students") OR TITLE-ABS-KEY (students)) AND (PUBYEAR > 1999)

"3000"[Date - Publication] NOT (review[Publication Type] NOT systematic[sb])	
Articles found at 03/07/25 = 11	Articles found at 03/07/25 = 26

3. What are the policies related to food systems, physical activity promotion and public education campaigns for the youth in Tanzania to address obesity?

**Pubmed**	**Scopus**
(obesity[MeSH Terms] OR overweight[MeSH Terms] OR "body mass index"[MeSH Terms] OR obesity[Title/Abstract] OR obese[Title/Abstract] OR overweight[Title/Abstract] OR "excess weight"[Title/Abstract] OR "weight gain"[Title/Abstract])AND (policy[Title/Abstract] OR policies[Title/Abstract] OR "public policy"[Title/Abstract] OR "health policy"[Title/Abstract] OR "public health policy"[Title/Abstract] OR "nutrition policy"[Title/Abstract] OR legislation[Title/Abstract] OR regulation[Title/Abstract] OR governance[Title/Abstract] OR "policy implementation"[Title/Abstract] OR "policy development"[Title/Abstract] OR planning[Title/Abstract] OR enforcement[Title/Abstract] OR taxation[Title/Abstract] OR tax[Title/Abstract] OR subsidy[Title/Abstract] OR subsidies[Title/Abstract] OR labeling[Title/Abstract] OR "food labeling"[Title/Abstract] OR marketing[Title/Abstract] OR "advertising restrictions"[Title/Abstract] OR "school policy"[Title/Abstract] OR "workplace policy"[Title/Abstract] OR "national plan"[Title/Abstract] OR "strategic plan"[Title/Abstract] OR strategy[Title/Abstract] OR strategies[Title/Abstract] OR framework[Title/Abstract] OR program[Title/Abstract] OR programs[Title/Abstract] OR initiative[Title/Abstract] OR initiatives[Title/Abstract] OR "health systems"[Title/Abstract] OR multisectoral[Title/Abstract] OR monitoring[Title/Abstract]) AND (Tanzania[MeSH Terms] OR Tanzania[Title/Abstract]) AND (Adolescent[MeSH Terms] OR "Young Adult"[MeSH Terms] OR Students[MeSH Terms] OR youth[Title/Abstract] OR "young people"[Title/Abstract] OR "young adults"[Title/Abstract] OR adolescents[Title/Abstract] OR teenagers[Title/Abstract] OR "university students"[Title/Abstract] OR "college students"[Title/Abstract] OR "secondary school students"[Title/Abstract]) AND ("2000/01/01"[Date - Publication] : "3000"[Date - Publication] NOT (review[Publication Type] NOT systematic[sb])	(TITLE-ABS-KEY (obesity) OR TITLE-ABS-KEY (obese) OR TITLE-ABS-KEY (overweight) OR TITLE-ABS-KEY ("body mass index") OR TITLE-ABS-KEY ("excess weight") OR TITLE-ABS-KEY ("weight gain")) AND (TITLE-ABS-KEY (policy) OR TITLE-ABS-KEY (policies) OR TITLE-ABS-KEY ("public policy") OR TITLE-ABS-KEY ("health policy") OR TITLE-ABS-KEY ("public health policy") OR TITLE-ABS-KEY ("nutrition policy") OR TITLE-ABS-KEY (legislation) OR TITLE-ABS-KEY (regulation) OR TITLE-ABS-KEY (governance) OR TITLE-ABS-KEY ("policy implementation") OR TITLE-ABS-KEY ("policy development") OR TITLE-ABS-KEY (planning) OR TITLE-ABS-KEY (enforcement) OR TITLE-ABS-KEY (taxation) OR TITLE-ABS-KEY (tax) OR TITLE-ABS-KEY (subsidy) OR TITLE-ABS-KEY (subsidies) OR TITLE-ABS-KEY (labeling) OR TITLE-ABS-KEY ("food labeling") OR TITLE-ABS-KEY (marketing) OR TITLE-ABS-KEY ("advertising restrictions") OR TITLE-ABS-KEY ("school policy") OR TITLE-ABS-KEY ("workplace policy") OR TITLE-ABS-KEY ("national plan") OR TITLE-ABS-KEY ("strategic plan") OR TITLE-ABS-KEY (strategy) OR TITLE-ABS-KEY (strategies) OR TITLE-ABS-KEY (framework) OR TITLE-ABS-KEY (program) OR TITLE-ABS-KEY (programs) OR TITLE-ABS-KEY (initiative) OR TITLE-ABS-KEY (initiatives) OR TITLE-ABS-KEY ("health systems") OR TITLE-ABS-KEY (multisectoral) OR TITLE-ABS-KEY (monitoring)) AND (TITLE-ABS-KEY (tanzania)) AND (TITLE-ABS-KEY (youth) OR TITLE-ABS-KEY ("young people") OR TITLE-ABS-KEY ("young adults") OR TITLE-ABS-KEY (adolescents) OR TITLE-ABS-KEY (teenagers) OR TITLE-ABS-KEY ("university students") OR TITLE-ABS-KEY ("college students") OR TITLE-ABS-KEY ("secondary school students") OR TITLE-ABS-KEY (students)) AND (PUBYEAR > 1999) AND NOT DOCTYPE (re)
Articles found at 03/07/25= 38	Articles found at 03/07/25= 88
