# Supplementary material for: Obesity in Tanzanian Youth (15–35 Years): From Nutrition Transition to Policy Action—A Scoping Review
Source: Nutrients. 2025 Dec 24;18(1):61. doi: 10.3390/nu18010061 (PMC12787505; doi:10.3390/nu18010061)
Supplement: Supplementary file 1 [file nutrients-18-00061-s001.zip › Addendum S2.pdf]

nutrients

MDPI

Supplementary Materials for

Youth Obesity in Tanzania: From Nutrition Transition to Policy Action—A Scoping Review

Addendum S2. Included studies after full-text screening

First author, year	Type of study	Population (n, age)	Context (rural/urban)	Summary of findings
Ahmed, Kedir Y. et al., 2020 [37]	Cross-sectional study	n=11738 women<br>Age:<br>15–24 years: 40.0%<br>25–34 years: 28.2%<br>35–49 years: 31.8%	Urban/Rural	Among the overall sample, overweigh and obesity prevalence was 18.4% and 10% respectively.<br>Among 15–24 years: Overweigh and obesity prevalence was 12% and 2.8% respectively.<br>Among 25–34 years: Overweigh and obesity prevalence was 21.9% and 11.9% respectively.<br>Reproductive age women who attained secondary or higher education (RRR = 1.48; 95% CI: 1.11, 1.96), those who resided in wealthier households (RRR = 2.31; 95% CI: 1.78, 3.03) and those who watched the television (RRR = 1.26; 95% CI: 1.06, 1.50) were more likely to be overweight. The risk of experiencing obesity was<br>higher among reproductive age women who attained secondary or higher education (RRR = 1.79; 95% CI: 1.23, 2.61), those who were formally employed (RRR = 1.50; 95% CI: 1.14, 1.98), those who resided in wealthier households (RRR = 4.77; 95% CI: 3.03, 7.50), those who used alcohol (RRR = 1.43; 95% CI: 1.12, 1.82) and/or watched the television (RRR = 1.70; 95% CI: 1.35, 2.13).
Amugsi, D.A. et al., 2017 [16]	Cross-sectional survey	N=5115 non-pregnant women<br>Age: 15–49 years.	Urban	Overweight prevalence was 14.1% in 1991, 20.5% in 1996, 18.9% in 2004 and 21% in 2009. Obesity prevalence was 3.6% in 1991, 7.8% in 1996, 9.7% in 2004, and 11.8% in 2009.
Darling, A.M. et al., 2020 [27]	Cross-sectional study	Tanzania urban: n=743<br>Age range<br>10-14 years: 66.7%<br>15-19 years: 33.3%	Urban/rural	The prevalence of overweight in urban Tanzania was 5.1%. The prevalence ratio of overweight in rural Tanzania was 1.39 (95% CI, 0.80, 2.42)

 https://doi.org/10.3390/nu18010061

First author, year	Type of study	Population (n, age)	Context (rural/urban)	Summary of findings
		Tanzania rural: n= 825<br>10-14 years: 32.3%<br>15-19 years: 67.7%		
Gibore, N.S. et al., 2023 [17]	Cross-sectional study	N=749, 42.1% males<br>Age (mean, SD): 47.6 ± 14.3 years	Urban	Overall, 63.5% (33.3% overweight and 29.9% obese) were overweight or obese, 4.5% were diabetic and 43.4% were hypertensive. Only 35.4% of participants had adequate knowledge of CVDs risk factors.
Gona, P.N. et al., 2021 [24]	Epidemiological analysis	GBD 2019 population-level estimates for Tanzania (no primary sample; modelled for entire national population, all ages)	Urban/rural	The age-standardized prevalence of obesity (BMI ≥ 30 kg/m²) in Tanzania showed a marked increase between 1990 and 2019. In adults aged 20 years and older, obesity prevalence in 2019 was estimated at 5.4 % among males (95 % uncertainty interval 4.4–6.5 %) and 12.8 % among females (95 % UI 11.2–14.6 %). This represents more than a doubling of obesity over the 29-year period for both sexes, with the rise being particularly pronounced in women.<br>Among children and adolescents aged 2–19 years, obesity prevalence in 2019 reached 5.4 % in boys (95 % UI 4.4–6.5 %) and 5.3 % in girls (95 % UI 4.3–6.5 %), again roughly doubling from the levels observed in 1990.
Ismail, A. et al., 2020 [28]	Cross-sectional survey	N= 1226, 44.7% males<br>Age (mean, SD): 13.7 (2.25)	Rural	Overweight and obesity affected 5.23% of participants. Girls had higher HAZs (b: 0.46, 95% CI 0.33, 0.59, P < 0.0001) and body mass index (BMI)-for-age-z-scores (BAZs) (b: 0.20, 95% CI 0.05, 0.35, P= 0.0098) than boys. Age was inversely associated with Height-for-age-z-scores (HAZs) (b: 0.13, 95% CI 0.17, 0.08, P < 0.0001) and BAZs (b: 0.05, 95% CI 0.10, 0.004, P= 0.0327).
Ismail, A. et al., 2023 [49]	Cross-sectional study	N=654, 57.8%<br>Age (mean, SD): 42.4±12.5 years	Rural/Urban	Higher food prices and lower diet quality persisted during the COVID-19 pandemic. Economic and social vulnerability and reliance on markets (and lower agriculture production) were negatively associated with diet quality. Although recovery was evident, consumption of healthy diets remained low.
Jones-Smith, J.C et al, 2011 [25]	Cross-sectional survey	N=17,021 women<br>Age range: 15–49 years	Urban/Rural	In just eight years, overweight has increased mainly in the wealthiest quintiles (from 30.3% to 42.8%), but also in the middle quintiles. In the poorest quintile, the increase was minimal.

https://doi.org/10.3390/nu18010061

First author, year	Type of study	Population (n, age)	Context (rural/urban)	Summary of findings
				The SII (slope index of inequality) went from -24.9 in 1996 to -39.7 in 2004, inequality increased sharply.
Keding GB. et al., 2021 [48]	Cross-sectional survey	n= 252 women<br>Age (years):<br>Range: 16-45<br>Mean ± SD: 33.3 ± 6.9	Rural	The five dietary patterns were "traditional- coast," characterized by fruits, nuts, starchy plants, and fish; "traditional-inland," characterized by cereals, oils and fats, and vegetables; "purchase," characterized by bread and cakes (usually fried in oil), sugar, and black tea; "pulses," characterized mainly by pulses, with few or no vegetables; and "animal products," characterized by a high consumption of meat, eggs, and/or milk. Significant positive associations were found, among others, between the purchase pattern and BMI (ϱ = 0.192, p = .005) and between the animal products pattern and wealth (ϱ = 0.168, p = 0.002).
Khamis, A.G. et al., 2021 [55]	Cross-sectional study	N=510, 52.7% females<br>Age median (IQR): 36 (52–25)	Urban/rural	The prevalence of general obesity based on BMI was 20.2% (95%CI; 16.9–23.9), abdominal obesity based on WHR was 37.8% (95%CI; 33.7–42.1), and WC was 29.1% (95%CI; 25.2–33.1). More than half (54.3%) of the participants consumed an adequate dietary diversity (DDS>4).
Lwabukuna, W.C. et al., 2021 [29]	Cross-sectional study	n=217, 32% males<br>Age:<br>Young adolescents (14-17 years): n=162 (75%)<br>Elder adolescents (18-19 years): n=55 (25%)	Urban	The prevalence of full-blown metabolic syndrome was 1.4% (3). Overall, the clinical markers included: dyslipidaemia 30% (64), central obesity 22% (48), hyperglycaemia 13% (29) and hypertension 2% (4). Prevalence of central obesity was 26% (42) among young adolescents and 11% (6) among elderly adolescents and the difference was significant (p value= 0.02).
Madzorera I., et al., 2021 [52]	Cross-sectional study	N= 868 women<br>Age (mean, SD): 31.5 (± 7.7) year	Rural	High prevalence of maternal overweight (24.3%) and obesity (13.1%)<br>Food crop diversity was positively associated with prime diet quality score (P< 0.001). For women living close (<1.1 km) to markets, producing 1 additional food crop was associated with a 0.67 (95% CI, 0.22–1.12) increase in prime diet quality score, versus a 0.40 (95% CI, 0.24–0.57) increase for women living farther away.

First author, year	Type of study	Population (n, age)	Context (rural/urban)	Summary of findings
Malliga, E. et al., 2013 [34]	Cross-sectional survey	N=833, 60% males<br>Age range: 15-65 years	Urban	When a Body Mass Index (BMI) comparison made between the genders females are exposed the uppermost, normal proportion of overweight/obese BMI (43.11%) than the male population (36.36%). Of the women, 15.59% and 29.05% of the men had hypertension (≥140/90 mmHg).
Mchau, G. et al., 2024 [35]	Cross-sectional study	N=44120 primary school adolescent<br>Age range:<br>10-14: 90.4%<br>15-19: 9.6%	Urban	The prevalence of anaemia was 34.1%, while stunting and overweight had a prevalence of 32% and 4.2%, respectively. Approximately 41.7%, 13.5%, and 0.3% had single, double, and triple burden malnutrition-related conditions, respectively. Females were found to have a higher risk of being overweight compared with males
Mgetta, N.J. et al., 2024 [30]	Cross-sectional study	N= 247, 53% males<br>Age range:<br>24-25 years: 62.8%<br>>25 years: 37.2%	Urban	Overweight prevalence was 21.8%, while obesity prevalence was 14%. University students are a vulnerable group in developing obesity/overweight due to the transitional stage. Being overweight and obese was associated with being female, increased age and being married. High dietary diversity was also linked with abdominal obesity.
Mosha, D. et al., 2021[38]	Cross-sectional survey	N= 1004 women<br>Age (mean, SD): 30.2 (±8.1) years.	Urban	Prevalence of overweight and obesity was high (50.4%), and underweight was 8.6%. The risk of overweight/obesity was higher among older women (35–49 vs 15–24 years: PR 1.59; 95% CI: 1.30–1.95); women of higher wealth status (PR 1.24; 95% CI: 1.07–1.43); and informally employed and married women. Attaining moderate to high physical activity (≥600 MET) was inversely associated with overweight/obesity (PR 0.79; 95% CI: 0.63–0.99). Dietary sugar intake (PR 1.27; 95% CI: 1.03–1.58) was associated with increased risk, and fish and poultry consumption (PR 0.78; 95% CI: 0.61–0.99) with lower risk of overweight/obesity.
Msemo, O.A. et al., 2018 [21]	Cross-sectional study	N= 2629 women<br>Age, median (range): 28.0 (18–40) years.	Rural	The age-standardised prevalences of pre-hypertension and hypertension were 37.2 (95% CI 34.0–40.6) and 8.5% (95% CI 6.7–10.8), respectively.<br>The prevalence of obesity was 5.25 among the overall sample, in particular, 32.6% in normotensive women, 51.5% in pre-hypertensive women and 15.9 in hypertensive women. In multivariate analysis, increasing age, obesity and haemoglobin levels were significantly associated with pre-hypertension and hypertension.

https://doi.org/10.3390/nu18010061

First author, year	Type of study	Population (n, age)	Context (rural/urban)	Summary of findings
Msollo, S.S. et al., 2025 [59]	Cross-sectional study	n=253, 49% males<br>Age: secondary school (not specified)	Urban	Only 20.2% (n = 51) and 43.5% (n = 110) reported consuming fruits and vegetables 7 days a week, respectively. Most of the participants (82.2%, n = 208) were aware of the causes of overweight and obesity, and increasing physical activity (51.8%, n = 131) was the most cited preventive measure. Being in a higher level of study were significantly associated with increased knowledge and practices on prevention of overweight and obesity.
Muhihi, A. et al., 2021 [46]	Cross-sectional survey	N= 97 men<br>Age (mean, SD): 31.6±6.4 years.	Urban	Obesity prevalence: 4.1%.<br>More than half (53.6%) of the participants had energy expenditure of ≥4,000 kcal/week.
Mushengezi, B. et al., 2014 [22]	Cross-sectional study	N= 582 adolescents, 52.1% boys<br>Age: (mean, SD) 16.5±1.8 years,	Urban	The proportion of adolescents with overfat or obesity was<br>22.2%. Systolic, diastolic and combined hypertension was present in 17.5%, 5.5%, and 4.0% respectively. In the total population mean body fat percent correlated positively with diastolic blood pressure and mean arterial pressure
Mwanri, A.W. et al., 2025 [54]	Cross-sectional study	N= 512 women of reproductive age<br>Age range:<br>15-25 years: 55%<br>26–35 years: 32%<br>>35 years: 13%	Rural	About 42% of the women had no formal education and about one in three women own a mobile phone. About 70% consumed vegetables while 33% consumed deep fried foods. Only 34% of the women met the minimum diet diversity (MDD-W) of five or more food groups. The mean NCD-protect score was 2.8 ± 1.4 and the NCD-risk score was 0.77 ± 0.97.
Nicholaus, C. et al., 2020 [43]	Cross-sectional study	n=164, 31.7% males<br>Age: mean (SD) 18.3(±0.7)	Urban/Rural	Mean intake of energy, vitamin C, iron, calcium, and zinc was 1392kcal, 24.8mg, 9.2mg, 134.5mg, and 4.3mg, respectively, which were below the Recommended Daily Allowance. Average carbohydrate, fat, and protein intake of 471.9g, 73.7g, and 80.7g, respectively, were slightly higher than the Recommended Daily Allowance in both sexes. Male had a significantly higher intake of protein and carbohydrates (P<0.001). Female had a significantly (P<0.001) high intake of fat compared to male adolescents. Overall, 23.1% of the adolescents were anaemic, 25% were overweight, and 6.1% were obese.

https://doi.org/10.3390/nu18010061

First author, year	Type of study	Population (n, age)	Context (rural/urban)	Summary of findings
Njiro, B.J. et al., 2023 [60]	Report	N=5528	Urban/Rural	In 2021, among a total of 2030 individuals screened for hypertension, 950 (46%) had high blood pressure; of these, about a third (31%) were newly diagnosed, and 15% were known hypertensive, either controlled on medications or uncontrolled. During the same period, we screened a total of 2026 individuals for diabetes; 10.1% of these had raised blood glucose, with newly diagnosed individuals comprising 3% of these. A total of 1472 people were also screened for obesity and about one third (29.6%) of the people did not meet the WHO-recommended 150 minutes of physical activity per week. Moreover, 35% of individuals reported not taking fruits and vegetables for five or more days per week. About 16% of females and 27% of males screened reported alcohol intake; with 5% of males reporting daily alcohol intake.
Nsanya, Mussa K. et al., 2019 [23]	Cross-sectional study	Tanzania<br>N=891, 58% males<br>Age (mean, SD): 19.2 (±3.1)	Urban	Overweight/Obesity prevalence: 12%<br>The overall prevalence of high blood pressure was 40%. The prevalence of prehypertension was 29% and that of hypertension was 11%. High blood pressure was independently associated with obesity, male sex, and among males aged above 20 years. Consumption of fruits/vegetables was associated with decreased odds for high blood pressure (aOR = 0.7, 95% CI: 0.50-0.98).
Nyangasa, M.A. et al., 2019 [19]	Cross-sectional study	N=470, 47.4% males<br>Age (mean, SD): 29±18 years	Urban/rural	The proportion of overweight/obese individuals was 26.4%. Obesity and hypertension significantly increased with age and were most prevalent in participants aged 45 years and above.
Nyaruhucha, C. N. M. et al., 2003 [20]	Cross-sectional study	N= 140<br>Sex not available<br>Age:<br>Age 19-50 years: 71.43%<br>Age 14-18 years: 28.57%	Urban	The prevalence of obesity among the sampled subjects in Morogoro Municipality was 25 %, whereby 15.7% had a Body Mass Index (BMI) of between 25 and 30, and 9.3% had a BMI of more than 30. Age and occupation of all the subjects, together with marital status of adult subjects, were significantly related with obesity status. Prevalence of obesity increased with the increased age whereby subjects in the 41-50 years had the highest rate (45.4%). Employed subjects had higher rate of obesity (22.2%) than pupils or students. Similarly, married adults had higher rate of obesity (27.8%) than the single ones (4.7%). Unlike the old age group (41-50 years), 70% of the youngest subjects were not aware about the harmful effects of obesity.

https://doi.org/10.3390/nu18010061

**First author, year**	**Type of study**	**Population (n, age)**	**Context (rural/urban)**	**Summary of findings**
Pallangyo, P. et al., 2020 [5]	Cross-sectional study	N=6691, 54.2% males<br>Age: 43.1 years (IQR: 18-95)	Urban	Obesity prevalence: 32.4%<br>Overweight: 34.8%<br>Factors that significantly associated with obesity were, age ≥ 40, being female, a current working status, habitual breakfast skipping, poor water intake, high soft drink consumption, regular fast-food intake, low vegetable and fruit consumption, alcohol consumption and hypertension.
Paul, E. et al., 2016 [36]	Cross-sectional study	N=9131 women<br>Age:<br>15-19 years: 22.7%<br>20-29 years: 32.7%<br>30-39 years: 25.9%<br>40-49 years: 18.7%	Urban/Rural	About 7.92% of the Tanzanian women of reproductive age were obese, 15% were overweight, and 11.5% were underweight. Women from Mainland Tanzania (6.56%) were significantly less likely (AOR = 0.66, 95% CI: 0.53–0.82) to be affected by obesity as compared to women from Zanzibar (12.19%).
Paulo, H.A. et al., 2022 [38]	Cross-sectional study	N=1004 non-pregnant women<br>Age: 30.2 (±8.1) years	Urban	Prevalence: 27.8% were overweight and 22.6% were obese. All 1004 women in the study consumed starchy staple foods. Of all the women studied, 10.5%, 1.7% and 3.8% consumed vitamin A rich dark green vegetables, nuts and seeds, and beans and peas, respectively. Compared with women in the lowest quintile of Prime Dietary Quality Score (PDQS), those who were in the highest quintile were significantly less likely to be overweight or obese (Adjusted Prevalence Ratio (APR) = 0.76, 95%CI: 0.62, 0.89) (F for trend = 0.029). Risk factors included the highest consumption of animal foods (APR = 2.81, 95% CI: 1.51–3.51) and fast food (APR = 2.57, 95% CI: 1.24–4.34).
Sarfo, J. et al., 2021 [51]	Cross-sectional and longitudinal study	N = 292 women<br>Age (mean, SD): 32.24±8.55	Rural	In Tanzania the overweight/obesity rate was 42%. Several patterns were identified, yet a "plant-based pattern" largely characterized by unprocessed and minimally processed foods and a "purchase pattern" mainly distinguished by highly processed foods were dominant. The "plant-based pattern" was inversely or not associated with overweight/obesity, while the "purchase pattern" had a positive association or no association.

**First author, year**	**Type of study**	**Population (n, age)**	**Context (rural/urban)**	**Summary of findings**
Shayo, Grace A. et al., 2011 [20]	Cross-sectional study	N=1249, 65.2% females<br>Age groups:<br>18-24 years: 26.4%<br>25-34 years: 33.1%<br>35-44 years: 20.1%<br>45-54 years: 11.5%<br>55+ years: 8.9%	Urban	The overall prevalence of obesity was 19.2% (240/1249). However, obesity was significantly more prevalent in women (24.7%) than men (9%), $p < 0.001$.
Temba, G.S. et al., 2025 [47]	Randomized controlled trial	N= 77 men<br>Age: 25.6 years (IQR: 21–27.2)	Rural/Urban	The switch from heritage-style to Western-style diet affected different metabolic pathways associated with noncommunicable diseases and promoted a pro-inflammatory state with impaired whole-blood cytokine responses to microbial stimulation.
Tengia-Kessy, A. et al., 2020 [31]	Cross-sectional study	N=400 secondary school girls<br>Age (mean, SD): 15.1±1.5 years	Urban	The proportion of adolescents with excess body weight (BMI >+1SD) was 23%. The majority (63%), reported unhealthy dietary habits while half (51.5%) of them had moderate level of knowledge on healthy eating.
Tluway, F.D. et al., 2018 [33]	Cross-sectional survey	N=619, 42.8% males<br>Age (mean, SD): 16.7±1.68 years	Semi-rural	The overall prevalence of overweight and obesity was 9.2% with more girls being overweight and obese than boys ($P<0.0001$).
Villamor, E. et al., 2006 [32]	Cross-sectional study	N=73689 women<br>Age: 14 –52 years	Urban	The prevalence of obesity rose steadily and progressively from 3.6% in 1995 to 9.1% in 2004 [adjusted prevalence ratio (PR): 1.97; 95% CI: 1.66, 2.33; P for trend for year 0.0001]. Underweight showed only a modest decline from 3.3% in 1995 to 2.6% in 2004 (adjusted PR: 0.91; 95% CI: 0.75, 1.10; P for trend for year 0.003), whereas no change was observed in the prevalence of wasting. In the most recent years (2003 and 2004), obesity was positively associated with age, parity, and socioeconomic status and inversely with HIV infection.
